# CD105^+^-mesenchymal stem cells migrate into osteoarthritis joint: An animal model

**DOI:** 10.1371/journal.pone.0188072

**Published:** 2017-11-30

**Authors:** Pablo Fernandez-Pernas, Iván Rodríguez-Lesende, Alexandre de la Fuente, Jesús Mateos, Isaac Fuentes, Javier De Toro, Fco J. Blanco, M. C. Arufe

**Affiliations:** 1 Grupo de Terapia Celular y Medicina Regenerativa (TCMR-CHUAC), CIBER BBN/ISCIII, Instituto de Investigación Biomédica de A Coruña (INIBIC), Complexo Hospitalario Universitario de A Coruña (CHUAC), SERGAS, Departamento de Ciencias Biomédicas, Medicina y Fisioterapia, Facultade de Oza, Universidade da Coruña (UDC), As Xubias, A Coruña, Spain; 2 Grupo de Investigación de Proteómica-PBR2-ProteoRed/ISCIII-Servicio de Reumatologia, Instituto de Investigación Biomédica de A Coruña (INIBIC), Complexo Hospitalario Universitario de A Coruña (CHUAC), SERGAS, Universidade da Coruña (UDC), As Xubias, A Coruña, España; Instituto Butantan, BRAZIL

## Abstract

Mesenchymal stem cells are being the focus of connective tissue technology and regenerative medicine, presenting a good choice cell source for improving old and well recognized techniques of cartilage defect repair. For instance, the autologous chondrocyte transplantation using new concepts of regenerative medicine. The present study investigated the risk of xenogenicity of human synovial membrane-derived MSCs, injected into the monkeys using intravenous and intra-articular administration.

The animal models used were adult monkeys Rhesus which had been injured into the left knee to create an Osteoarthritis (OA) animal model. CD105^+^-MSCs were injected twice into the OA monkeys with an interval of one week between them. The animals were euthanized one month after treatment. Immunohistochemistry analysis of different organs: spleen, heart, fat, liver, gut, pancreas, lung, skeletal muscle and kidney from the animals revealed that CD105^+^-MSCs migrated towards the injured knee joint. MSCs naive were found statistically significant increased in the injured knee in front of healthy one. CD105^+^-MSCs were negatives for CD68 and the area where CD105^+^-MSCs were found presented SDF-1 increased levels in front of healthy knee. We concluded that a characterized MSCs subset could be a safe alternative for cell therapy in clearly localized pathologies.

## Introduction

Mesenchymal stem cells (MSCs) are an attractive cell source for cartilage regenerative medicine since they can be extracted in a minimally invasive manner, they are easily isolated and expanded and they are able to differentiate towards several mesenchymal lineages, including chondrogenesis [1]. Therefore, in the last twenty years MSCs are being the focus of connective tissue technology and regenerative medicine, presenting a good choice cell source for improving old and well-established techniques of cartilage defect repair. For instance, MSCs are also being tested as an perfect cell source in combination with newly developed implantable scaffolds or as a target/carrier cell in other new concepts of regenerative medicine [2].

Last decade, MSCs move on like a good choice cell source to donor-derived chondrocytes and several clinical studies are currently on trial[3, 4]. Osteoarthritis (OA) Cynomolgus monkey model have been studied by Ham et al[5] and there are several studies about the efficacy of different hormone treatments on OA process using also this animal model[6].

The present study investigated the risk of xenogenicity of human synovial membrane-derived MSCs, injected into the monkeys using intravenous or intra-articular administration, through checking CD68 expression by immunofluorescence and discard that MSCs injected could have been destroyed by macrophages. We were interested in localizing the tissues where the labelled DiO-CD105^+^-MSCs, DiI-CD105^+^-MSCs or GFP-CD105^+^-MSCs injected in the animals were able to migrate. SDF-1 is a powerful chemo-attractant cytokine that promotes directional migration of hematopoietic and non-hematopoietic cells [7] and it also was tested to find out whether it was involved in the behaviour of MSCs injected.

## Material and methods

### Tissue collection

Normal synovial membranes were obtained from 10 patients undergoing knee amputation who had no history of joint or inflammatory disease. All tissues were obtained with fully informed and signed consent from all subjects and ethical approval under the supervision of Ethical Committee of Clinical Investigation of Galicia. All the methods were carried out in accordance with the approved guidelines of Spanish law (14/2007 and 1716/2011) of Spanish Biomedical Investigation.

#### Isolation, culture and characterizing of MSCs by fluorescence-activated cell sorting (FACS)

Synovial membranes from intact areas of clinically normal joints were harvested and subjected to sequential digestion using 1.2 U/ml dispase and 112 U/ml type I collagenase to isolate MSCs, as previously described[1, 8]. Monolayer cultures of MSCs from synovial membrane were cultured in Dulbecco´s Modified Eagles Medium (DMEM), 15% v/v foetal bovine serum (FBS), 1% v/v penicillin and 1% v/v streptomycin (all from SIGMA-ALDRICH, Missouri, USA), when the cells lead 90% of confluence in culture they were separated into CD-105^+^sub-population by FACSAria cytometry sorter (BD Bioscience, Madrid, SP) using an antibody against anti-human CD-105 (BD, Pharmigen), which dilution was 1:20 of antibody each 1x10^6^ cells in 200 μl of PBS. The CD-105^+^sub-population were characterized by flow cytometry. The primary antibodies used were mouse anti-human CD34 (1:20 DakoCytomation, Barcelone, SP), FITC mouse anti-human CD45 (1:20), FITC mouse anti-human CD105 (1:100 from Serotec, Bavaria, GER), FITC mouse anti-human CD44 (1:100 from Serotec, Bavaria, GER) and PE-Cy5-conjugated mouse anti-human CD90 (1:20 from BD Pharmagen, Madrid, SP). Flow cytometry data were generated on CellQuest and DIVA software (BD Bioscience, Madrid, SP).

### Labelling of CD105^+^ -MSCs

Carbocyanine membranes probes have been used to label the CD105^+^sub-population. Carbocyanine dyes are extensively used as nontoxic labels for live-cell membranes and for following cell division by flow cytometry [9]. The octadecyl (C18) indocarbocyanines which acronym is DiI and its fluorescence emission is 670nm, was used to label the CD105^+^sub-population injected through the vein. The oxacarbocyanine which acronym is DiO and its fluorescence emission is 510nm, was used to label the CD-105^+^sub-population injected directly in the knee. In summary, 1mg/mL was diluted in Hank´s balanced salt solution (HBSS). The cells were incubated in 1μM of the stock solution for 5 minutes at 37°C and for 15 minutes at 4°C and the last were washed with phosphate-buffered saline (PBS) and diluted in saline to inject. The Lenti-^TM^ Lentiviral expression System (Clontech Laboratories Inc.) was used following the manufacturer´s to transfect LVX-GFP-puro into CD105^+^-MSCs and the cells were selected by incubating the cells in growth media supplemented with 1μg/mL puromycin (Clontech Laboratories Inc.)[10].

### Non human primate model

Adult male Cynomolgus monkeys (*Macaca Fascicularis*) were purchased from R C Hartelust BV (Tilburg, NL). They remain at Veterinarian Facility at least 90 days, before entering study, as a period of quarantine and adaptation. They are identified by tattoo on the chest and microchip. All animals weighing 10±1 kG each were housed together at Veterinarian Facility-accredited animal facility in a common room equipped with a reverse-filtered air barrier (10 air changes per hour, 100% fresh air) and full-spectrum light (8:00 AM to 8:00 PM) and conditioned to 23°C with relative humidity of 60%. The common room is provided with environmental enrichment (swings, hammocks, stairs, ropes, balls, bathtubs) as well as sensory stimulation (music and contact with caregivers), so the animals were entertained and thus reduced their aggressiveness. Animals were fed commercial primate chow supplemented with fresh fruit and were provided tap water ad libitum. Nine male adult animals (*Macaca Fascicularis*) were used in the experiments. Before being included in the study, hematological and biochemical controls, immunological evaluation and a general examination of the state of the animal were performed. Total duration of all experiments was 12 months.

Animals were transferred to the treatment room, where they will remain during the whole procedure, performing three weekly checks. The preparation for surgery started with a fasting period of 12 hours. Anaesthesia with Ketamine (K) plus Medetomidine (M): (K) 5–7.5 mg / Kg + (M) 0.033–0.75 mg / Kg IM was used. It is proceed to weighing and shaving the surgical area. The animals were transferred to the operating room where they were catheterized by the cephalic vein of both arms and an arterial route in radial arteria. An intravenous bolus muscle relaxant (atracurium 0.5–2.5 mg / k) was administered to perform endotracheal intubation. Inhalation anaesthesia was maintained with 2% sevoforane, oxygen flow 10–15 ml / k / min, and positive pressure mechanical ventilation. Before starting the procedure analgesics were administered and maintained the whole procedure (Fentanyl 0.01 mg / k / h + Remifentanil: 0.15 ugr / K / min = 9 ugr / k / H). 15 minutes before the end of the procedure, butorphanol (0.05–0.4 mg / Kg SC or IM) was administered as analgesic and 5 minutes after administration, continuous remifentanil infusion was suppressed.

After shaving and disinfecting the knee with chlorhexidine alcohol, it was performed a nail parapatellar incision. Next, patella was dislocated and a chondral defect of 4 mm in diameter was created in the zone of maximum load (maximum thickness from articular cartilage). Each chondral lesion will be covered with an autologous periosteum patch of 6–8 mm diameter, forming a cavity comprising or defect. This periosteum will proceed from the proximal tibia and was sutured continuously with a 9–0 suture. To secure or patch or defect, fibrin was used. Finally, the knee was sutured in layers: synovial membrane and fibrous capsule, with 6–0 Tricon; skin with 3–0 Novofil. Animals will only be isolated in the first postoperative day until cicatrisation. After that, they will be debited to the common primate room.

Animals were organized into 4 study groups. The defect cavity was filled with 500 μL suspension of 10 x 10^6^ DiO-CD105^+^-MSCs in physiological saline solution in the group 1(n = 2). The defect cavity was filled with 500 μL suspension of 10 x 10^6^ DiI-CD105^+^-MSCs in physiological saline solution and intravenous injection with 10 x 10^6^ DiO-CD105^+^-MSCs in group 2 (n = 2). The defect cavity was filled with 500 μL suspension of 10 x 10^6^ DiI-CD105+-MSCs in physiological saline solution and intravenous injection with 10 x 10^6^ GFP-CD105+-MSCs in group 3 (n = 2). The defect cavity was filled with 500 μL of physiological saline solution and intravenous injection with 10 x 10^6^ GFP-CD105^+^-MSCs in group 4 (n = 3) (Table 1).

**Table 1:** Groups of animals and treatments. Intra-articular (IA) injection. Intravenous (IV) injection.

Groups	Animals (N)	IA	IV
1	2	DiO-105^+^-MSCs	—
**2**	2	DiI-105^+^-MSCs	DiO-105^+^-MSCs
**3**	2	DiI-105^+^-MSCs	GFP-105^+^-MSCs
**4**	3	—	GFP-105^+^-MSCs

Body temperature, weight changes or abnormal behaviour were specific criteria used to determine the humane checkpoint when animals should be euthanized. In order to ensure animal welfare during the procedure, an animal monitoring protocol is included which allows corrective measures of suffering to be taken with the use of painkillers or slaughtering of the animal for humanitarian reasons. The monitoring protocol used was the one proposed by Morton and Griffiths [11].

All the animals were euthanized one month after the last injection and their tissues were frozen and included in paraffin to be analyzed furthermore by immunohistochemistry and immunofluorescence techniques respectively. The euthanasia method used was intramuscular anaesthesia with Ketamine (K) + Medetomidine (M): (K) 5–7.5 mg / kg + (M) 0.033–0.75 mg / kg and once the animal had slept, it was canalized thee cephalic vein, an anaesthetic agent (thiopental IV) and later potassium chloride were administered by rapid intravenous administration, which resulted in fibrillation and cardiac arrest.

All the methods were carried out in accordance with the approved guidelines of Spanish law (1201/2005). All experimental protocols were approved by Animal Ethical Committee of Galicia.

### Biochemical and serologic analysis

The biochemical and hematologic analysis made to the animals before and just after sacrificing consisted to extraction blood from the vein and hematologic parameters as RBC, haemoglobin, haematocrito, WBC, platelets, lymphocytes and neutrophils were made using Veterinarian-MS9 (Kemia Scientific). Serum from whole blood was measured for glucose, bun-urea, creatinine, total protein, albumin, amylase, calcium, phosphate, sodium, potassium, chloride, cholesterol, bilirubin, AST, ALT, alkaline phosphatase and triglycerides using Vitalab Selectra-Flexor (ATOM).

### Histological and immunofluorescence analysis

Representative tissues from each monkey were frozen in OCT embedding matrix (BDH Chemicals, Poole, UK) and in paraffin until their posterior analysis. Full-depth sections (thickness 4 μm) were cut with cryostat or microtome and fixed in 4% (w/v) paraformaldehyde in PBS, pH 7.6. Some sections were stained with haematoxylin and eosin (SIGMA-ALDRICH, Missouri, USA) to evaluate the distribution of cells and some sections were stained with Safranin O (all from SIGMA-ALDRICH, Missouri, USA) to evaluate the distribution of proteoglycans. Other sections were immunostained with antibodies: FITC mouse anti-human CD105 (1:100 from Serotec, Bavaria, GER), PE-Cy5-conjugated mouse anti-human CD90 (1:20 from BD Pharmagen, Madrid, SP), FITC mouse anti-human CD44 (1:100 from Serotec, Bavaria, GER), PE-conjugated mouse anti-human CD68 (1:50; from DakoCytomation) and PE-conjugated mouse anti-human SDF-1 (1:100; Sta. Cruz Biotechnology). For detection of primary antibodies do not directly labelled, the cells were washed with PBS, then incubated with Polyclonal rabbit anti-mouse IgD/PE Rabbit F(ab´)2 (1:1000 from DakoCytomation) for 30 min at room temperature. Negative control staining was performed using a FITC-conjugated mouse IgG1k isotype, a PE -conjugated mouse IgG1k isotype and PE-Cy5-conjugated mouse IgG1k isotype (all BD Pharmigen). The pictures shown were done by an Olympus microscopy. The highest magnification pictures (60x) were done with a confocal microscopy (A1R Nikon).

### Densitometry analysis

AnalySIS Image Processing (Soft Imaging system GmbH V. 5.0, Olympus, Münster, Germany) was used to do a densitometry quantification of the fluorescence signal obtained by immunofluorescence analysis shown in the pictures. Three fields 20 μm^2^ in size from each inmunofluorescence- FITC, PE and PE-Cy5- and time studied were quantified using arbitrary units for immunofluorescence values provided by the computer program. Values expressed as percentage of positive stain for each fluorochrome was normalized by DAPI signal.

### Statistics analysis

All analysis was performed in triplicate and one representative set was chosen to be shown. Comparisons between injection methods through labelled MSCs were done. To given the low number of animals used in each group no-parametric statistical analysis by one-way Anova was performed between groups of animals injected with different labelled MSCs (DiO, DiI and GFP) against the injection method. No-parametric statistical analysis by Mann Whitney and Kruskall-Wallis tests was done to compare the densitometry results from immunofluorescence analysis. SPSS 16.0 computer program was used to do the statistics analysis. A *p* value less than 0.05 was considered statistically significant.

## Results

### Isolation, characterization and labelling of CD105^+^-MSCs

**Fig 1:**
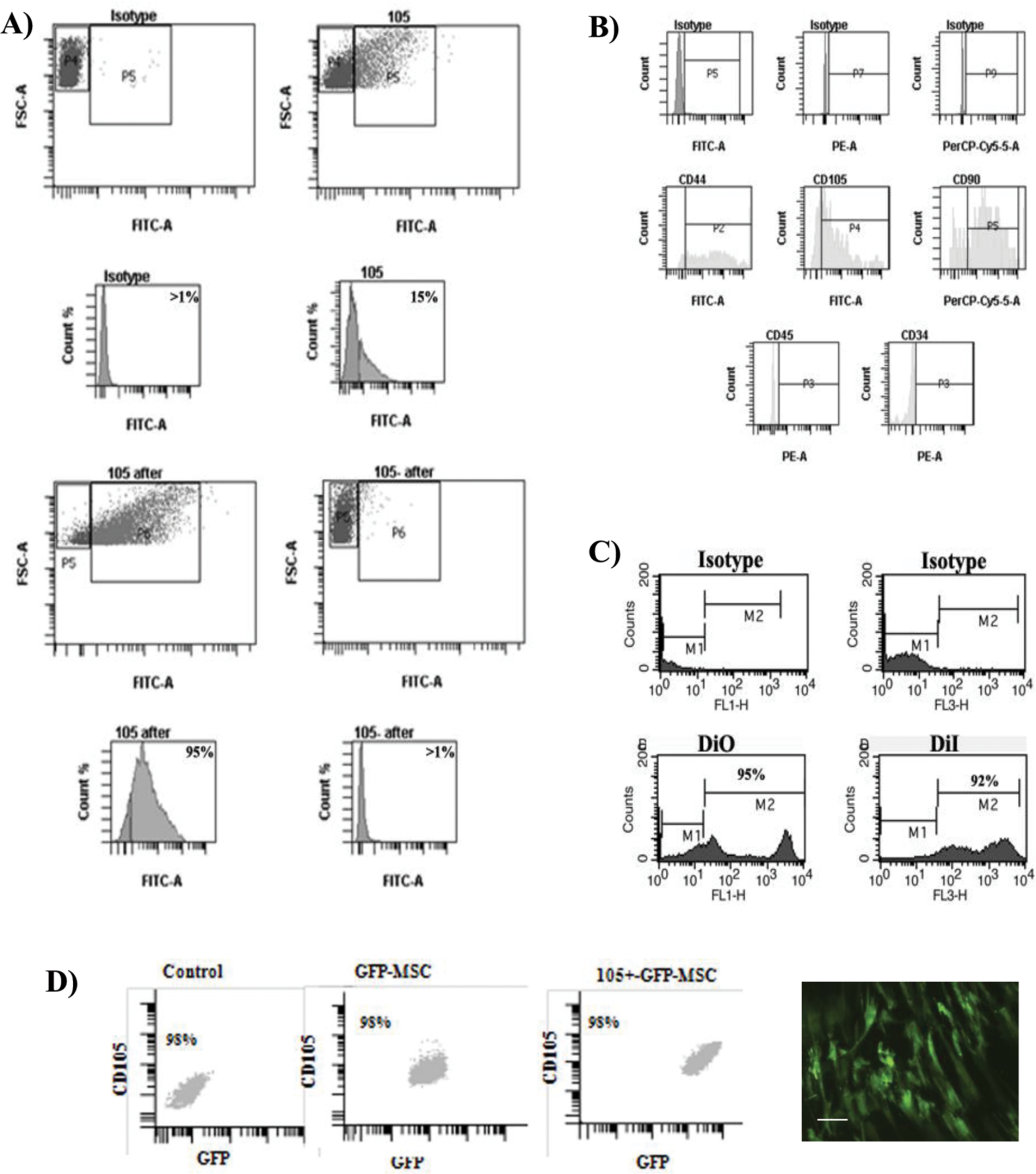
Characterization of CD105^+^-MSCs used in the cell therapy. A) Selective enrichment of CD105^+^ expressing cells. Fluorescence-activated cell sorting (FACS) analysis of the subpopulations of CD105^+^ pre-sorted (on the top) and post-sorted (on the bottom) from synovial membrane. 105 after was subpopulation of CD105^+^ post-sorted (on the left bottom) and 105- after was subpopulation of CD105^-^ post-sorted (on the right bottom). B) Characterization by flow cytometry analysis of the CD105^+^-MSC population using MSCs markers, CD44, CD105, CD90 and haematopoietic markers CD45 and CD34. C) Flow cytometry of subpopulation of CD105^+^-MSC labelled with oxacarbocyanine (DiO) and octadecyl (C18) indocarbocyanine (DiI) respectively. D) Characterization of GFP-CD105^+^-MSCs used to validate previous data obtained using DiO- CD105^+^-MSCs and DiI-CD105^+^-MSCs in animal model. Fluorescence-activated cell sorting (FACS) analysis of the MSCs populations (on the left), GFP-MSCs (on the middle) and post-sorted for CD105^+^ (on the right) from synovial membrane. Representative image of GFP-CD105^+^-MSCs in a plate before injection, bar represents 20 μm.

The percentage of CD105 positive cells in the MSC population was between 15–20% before separation, increasing at 95% following separation by FACSAria equipment. FACS analysis of CD105^+^-MSCs from synovial membranes after sorting showed at 95% accurate (Fig 1A). Phenotypic characterization of separated CD105^+^-MSCs showed no expression levels of CD45 and CD34 and high percentage of CD105 (95%), CD90 (99%) and CD44 (80%) (Fig 1B).

The accurate of DiI and DiO labelled cells were checked using flow cytometry by FACsScalibur directly on the cells, obtaining 92% of the cells positive for DiI and 95% of the cells positive for DiO (Fig 1C) at these concentrations. All CD105^+^-GFP-MSCs injected were positive for GFP (Fig 1D).

### Immunohistochemistry and immunofluorescence analysis

**Fig 2:**
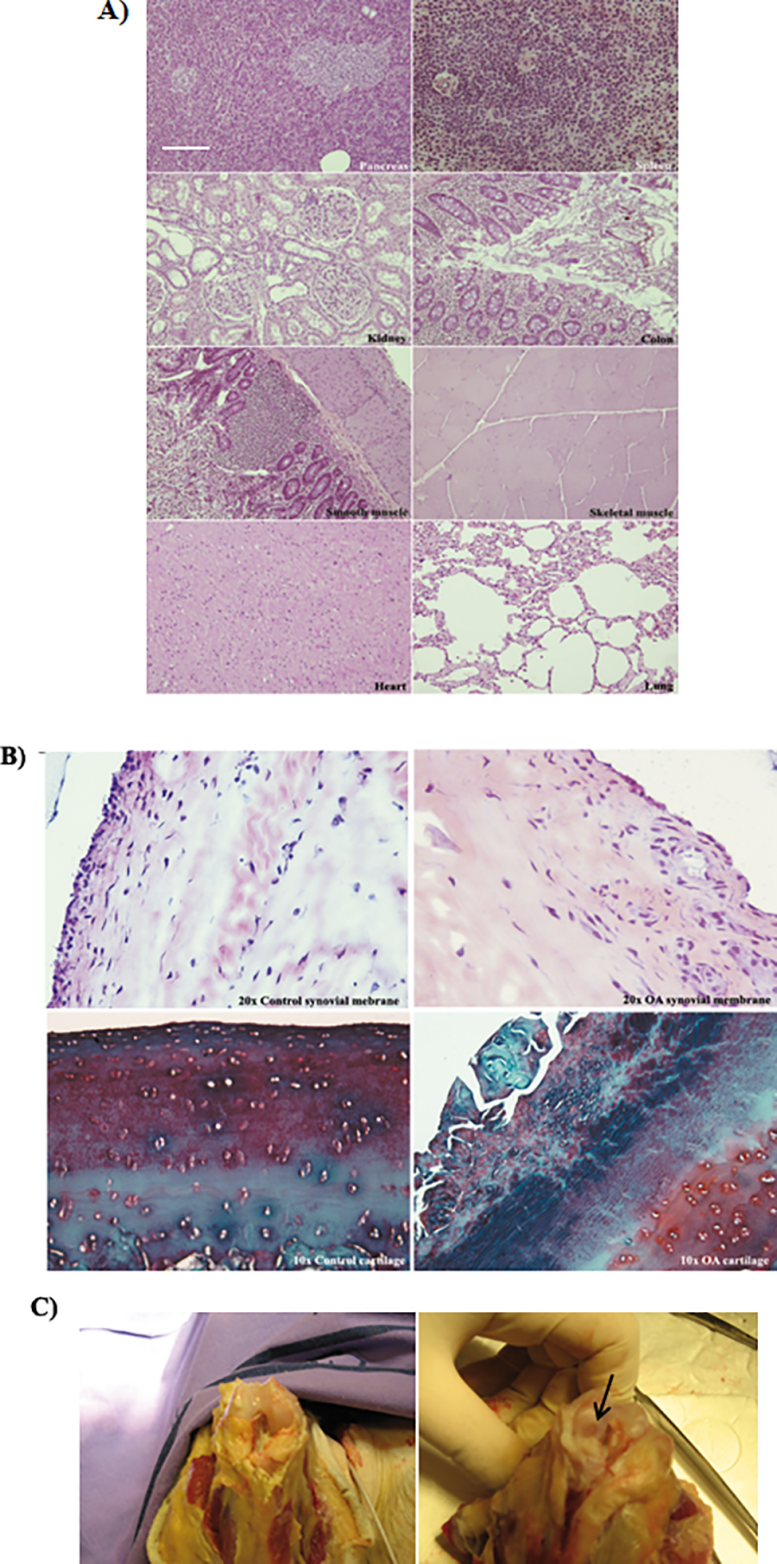
Osteoarthritis Cynomolgus monkey model. A) Histological appearance of pancreas, spleen, kidney, colon, smooth and skeletal muscle, heart and lung tissues from animals euthanized after MSCs injections. Sections at 4μm embedded in paraffin stained with haematoxylin and eosin (H-E) were shown. All pictures have the same magnification, bar represents 20 μm. B) Microscopic study form health and injured knees from Cynomolgus monkey model after sacrificing them. Synovial membrane and cartilage sections from animals of the study were shown. Histological appearance of tissues at 4μm embedded in paraffin to keep their consistency. Synovial membrane stained with haematoxylin and eosin (H-E) (magnification 20x) from right knee which was healthy on the left and left knee which was injured to provoke OA pathology on the right (up). Cartilage was stained with Safranine O (magnification 10x) from right knee which was healthy on the left and left knee which was injured to provoke OA pathology on the right (bottom). C) Macroscopic study from health and injured femoral condyles from Cynomolgus monkey model after sacrificing them. The arrow points lesion area.

All organs tested presented healthy morphology by haematoxylin-eosin stain histology analysis (Fig 2A). The hematologic (Table 2) and biochemical (Table 3) analysis of the animals before and after sacrificing revealed that they were healthy and their metabolic functions worked correctly. The synovial membrane from injured knee, which is the left one, was hypertrophy and destruction of cartilage and lack of proteoglicans was observed (Fig 2B on the right). Furthermore, no hypertrophy was observed in the synovial membrane from the right knee where normal structure of cartilage was observed in the healthy knee (Fig 2B on the left). Macroscopic visual analysis showed lack of cartilage in the injured knee (Fig 2C on the right) and no damage was observed in the healthy knee (Fig 2C on the left).

**Table 2:** Haematology analysis results from animals before and after euthanizing.

	Before euthanizing	After euthanizing
**RBC (total**	5,07 10^6^/μL-5,58 10^6^/μL	5,63 10^6^/μL±5,5810^6^/μL
**Haemoglobin**	12,6 mG/dL-2,7 mG/dL	12,2 mG/dL±2,9 mG/dL
**Hematocrit**	37,5%-46%	36,6%-45%
**WBG**	13,74 10^3^/ μL-11,90 10^3^/ μL	11,48 10^3^/ μL-12,84 10^3^/ μL
**Neutrophils**	63,7%-67,7%	63,7%-66,7%
**Lymphocytes**	25,6%-32,2%	24,2%-32%
**Platelets**	344 10^3^/ μL-340 10^3^/ μL	339 10^3^/ μL-341 103/μL

**Table 3:** Biochemistry analysis results of animals before and after euthanizing.

		Before euthanizing	After euthanizing
**Volume of blood**	mL	5	5
**Glucose**	mG/dL	106–76	91–76
**BUN-UREA**	mG/dL	38–55	41–43
**Creatinine**	mG/dL	1.11–1.06	0.64–0.93
**Total protein**	G/dL	6.84–7.18	6.65–7.06
**Albumin**	G/dL	3.75–3.63	3.63–3.55
**Calcium**	mG/dL	8.8–9.08	9.08–8.93
**Phosphate**	mG/dL	4.78–2.87	2.87–4.31
**Sodium**	meq/L	152–151	151–144
**Potassium**	meq/L	4.33–3.84	3.84–3.25
**Chloride**	mmol/L	111–119	119–113
**Cholesterol**	mG/dL	73–93	93–90
**Bilirubin**	mG/dL	0.69–0.44	0.44–0.61
**AST**	Ul/L	42–47	47–54
**ALT**	Ul/L	29–37	37–29
**Alkaline phos.**	Ul/L	116–97	128–160
**Triglycerides**	mg/dl	108	136

### Intra-articular injection

**Fig 3:**
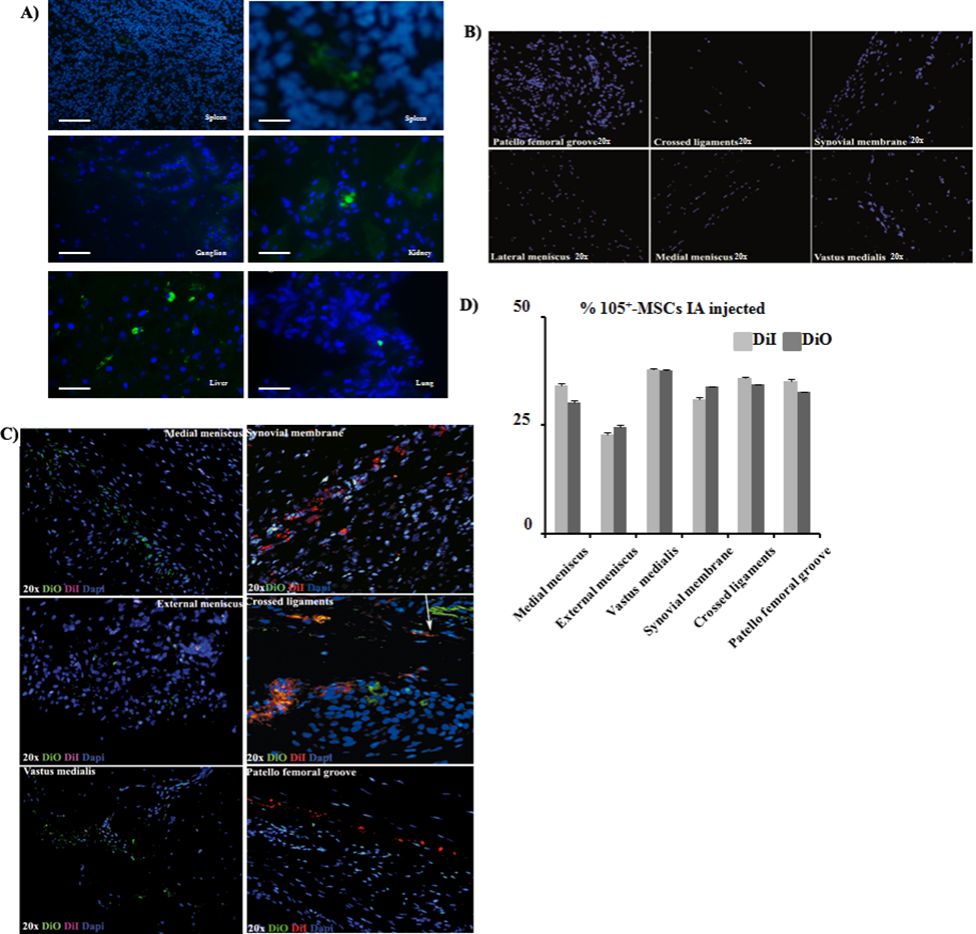
Immunofluorescence study from animal organs. A) Immunofluorescence analyses of spleen, ganglion, kidney, liver and lung from animals scarified after MSCs injections. All pictures have the same magnification, bar represents 20 μm except spleen on the right which bar represents 10 μm. B) Immunofluorescence analysis from healthy right knee from animals euthanized after MSCs injections. All pictures have the same magnification, bar represents 20 μm. C) Immunofluorescence analysis of sections of patella femoral groove, crossed ligaments, synovial membrane, internal meniscus, external meniscus and knee muscle (vastus medialis) of the animals injected IA with DiO-CD105^+^-MSCs and IV with DiI-CD105^+^-MSCs. All the sections are from the left knee which was injured and it was taken as OA (magnification 20x). The arrow points DiO-CD105^+^-MSCs and DiI-CD105^+^-MSCs very close between them. D) Histogram showed percentage of DiO and DiI positives signal normalized with DAPI signal from animals injected IA. AnalySIS Image Processing was used.

No DiI-CD105^+^-MSCs neither DiO-CD105^+^-MSCs injected IA were found them in heart, skeletal muscle or smooth muscle (Fig 3A and 3B).

Healthy joints. Microscopy analysis by immunofluorescence approach using an Olympus microscope showed no DiI-CD105^+^-MSCs neither DiO-CD105^+^-MSCs in the healthy knees from the animals injected with labelled cells (Fig 3B).

OA joints. DiI-CD105^+^-MSCs and DiO-CD105^+^-MSCs, injected via IA, were found in medial and lateral meniscus, skeletal muscle (*vastus medialis*), synovial membrane, crossed ligaments and patella femoral groove of injured joint knee (Fig 3C). Less than 50% of injected cells were found in the injured knee from the animals (Fig 3D).

### Intravenous injection

**Fig 4:**
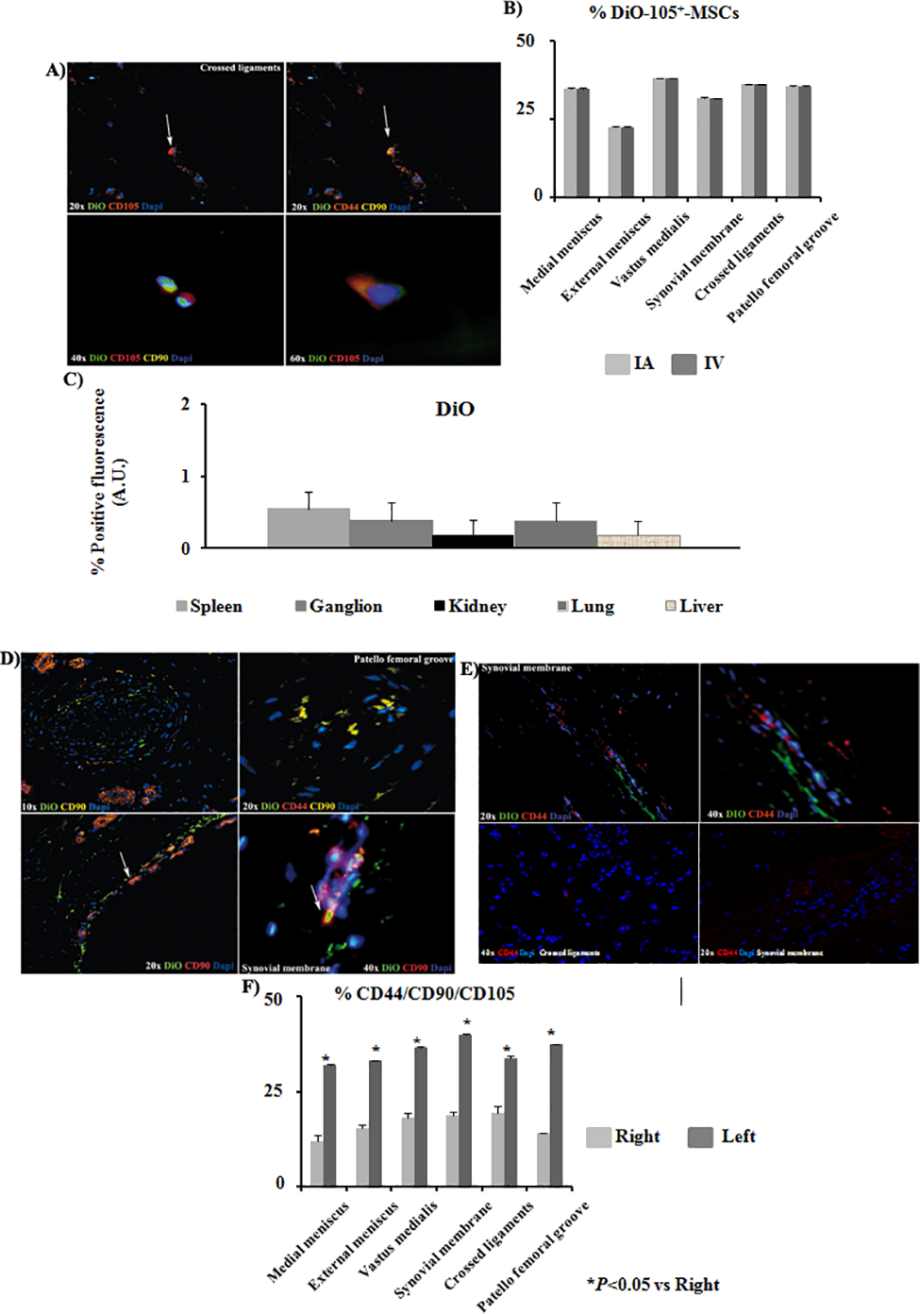
DiO-CD105^+^-MSCs migration study. A) Co-localization of DiO-CD105^+^-MSCs with antibodies against anti-CD105, anti-CD44 and anti-CD90 in crossed ligaments sections of left knee from animals injected with DiO-CD105^+^-MSCs IV (magnification were 20x, 40x and 60x). Arrows point cells which co-localized for CD44 and CD90 antibodies. B) Histogram showed percentage of DiO positives signal normalized with DAPI signal from animals IA injected in front of animals IV injected. AnalySIS Image Processing was used. C) Quantitative analysis to determine levels of positive DiO fluorescence in front of DAPI signal was done by AnalySIS Image software from tissues where DiO-CD105^+^-MSCs were found. D) Immunofluorescence analysis of sections from patella femoral grove and synovial membrane from the left knee at different magnifications (10x, 20x and 40x) from animals injected IA with DiO-CD105^+^-MSCs. Arrows point the same group of cells which co-localized for CD105, CD44 and CD90 antibodies. E) Immunofluorescence analysis of sections from synovial membrane at 20x and 40x magnifications from right knee (up) and left knee (down). F) Histogram showed percentage of CD44, CD90 and CD105 positives signal normalized with DAPI signal from animals injected IA with DiO-CD105^+^-MSCs. AnalySIS Image Processing was used. *P value less than 0.05 was considered statistically significant by Mann Whitney and Kruskall-Wallis.

Co-localization of CD105, CD44 and CD90 antibodies in the DiO-CD105^+^-MSCs IV injected were done by immunofluorescence. The results indicated that the patella femoral groove, synovial membrane and crossed ligaments from injured knee of the animals injected IV with DiO-CD105^+^-MSCs were also positives for CD90 and CD44 antibodies (Fig 4A). Furthermore densitometry analysis was done between animals injected IA versus IV with DiO-CD105^+^-MSCs (Fig 4B) observing no differences found regardless the injection form. Only less than 1% of DiO-CD105^+^-MSCs injected IV were detected in ganglion, spleen, lung, kidney and liver (Fig 4C). Naive MSCs positives for CD44, CD90 and CD105 were found statistically significant increased in injured knee in front of healthy one (Fig 4D, 4E and 4F).

**Fig 5:**
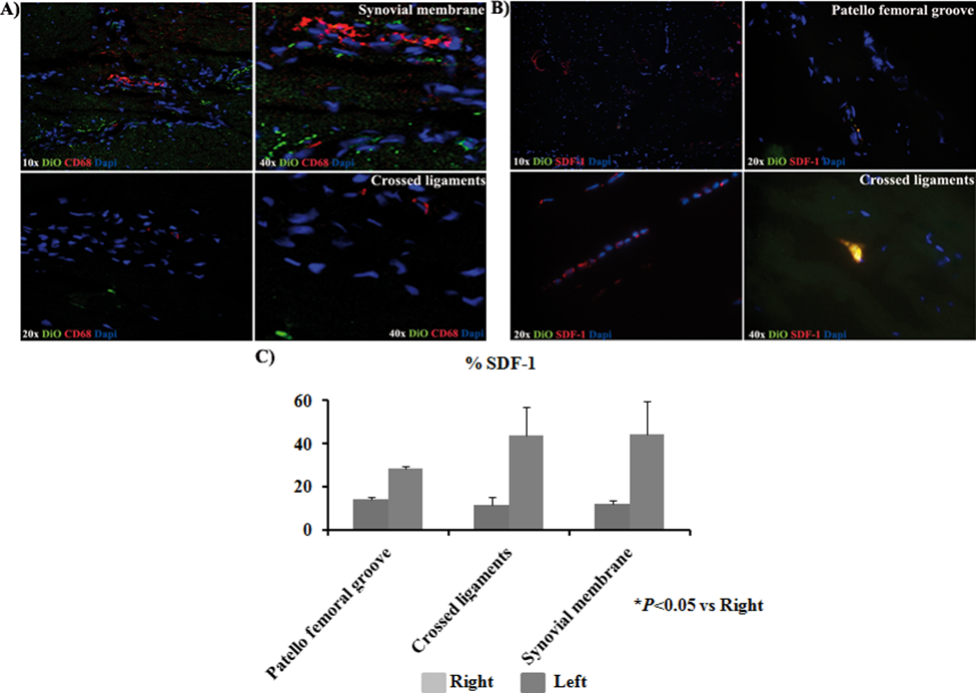
Study of DiO-CD105^+^-MSCs. A) Immunofluorescence analysis for CD68 of sections from synovial membrane and crossed ligaments of the animals IV injected with DiO-CD105^+^-MSCs expressing cells. All the sections are from the left knee which was injured to induce OA pathology (magnification were 10x, 20x and 40x). B) Immunofluorescence analysis for SDF-1 of sections from synovial membrane and crossed ligaments of animals IV injected with DiO-CD105^+^-MSCs expressing cells. All the sections are from the left knee with OA pathology (magnification were 10x, 20x and 40x). C) Histogram showed percentage SDF-1 positives signal normalized with DAPI signal from knee sections of animals group IA injected with DiO-CD105^+^-MSCs and knee sections from animals group IV injected with GFP-CD105^+^-MSCs. AnalySIS Image Processing was used. *P value less than 0.05 was considered statistically significant by Mann Whitney and Kruskall-Wallis.

**Fig 6:**
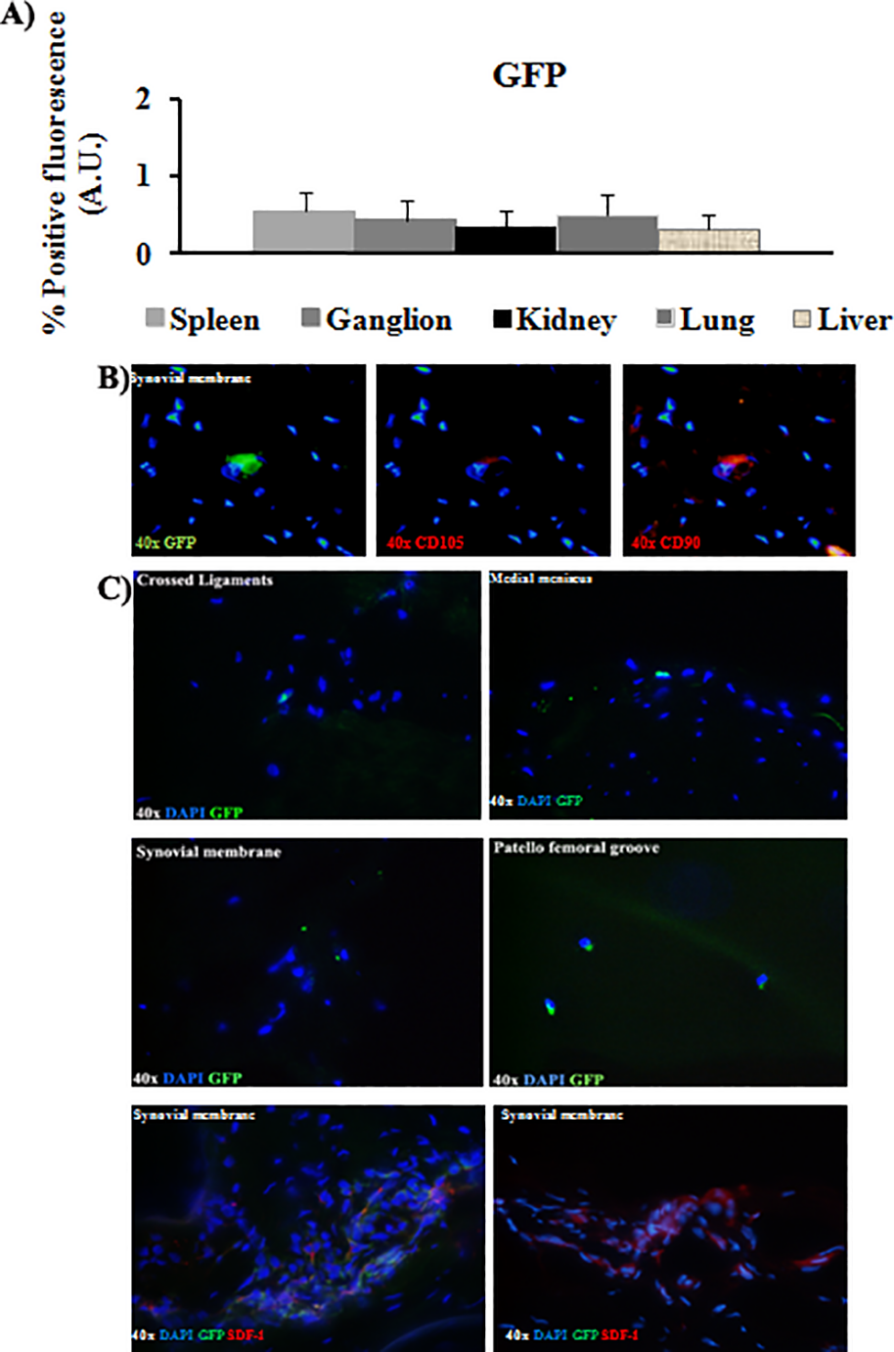
GFP-CD105^+^-MSCs migration study. A) Quantitative analysis was done by AnalySIS Image software from organs where GFP-CD105^+^-MSCs were found to determine levels of positive GFP fluorescence in front of DAPI signal. B) Co-localization of GFP-CD105^+^-MSCs with antibodies against anti-CD105 and anti-CD90 in synovial membrane sections of left knee from animals IV injected with GFP-CD105^+^-MSCs. All pictures have same magnification 40x. C) Representative pictures of GFP-CD105^+^-MSCs from crossed ligaments, synovial membrane, medial meniscus, and patella femoral groove and immunofluorescence analysis for SDF-1 of sections from synovial membrane from animals IV injected with GFP-CD105^+^-MSCs. All the sections are from the left knee with OA pathology (magnification 40x).

Immunofluorescence for CD68 and SDF-1 were done in synovial and crossed ligaments from injured knee of the animals injected IV with DiO-CD105^+^-MSCs. The results indicated that CD68 cells no co-localized with DiO positive cells (Fig 5A). Only SDF-1 positive cells were found in tissues from the injured knee and some of them co-localized with DiO-CD105^+^-MSCs (Fig 5B). Less than 1% of GFP-CD105^+^-MSCs injected IV were detected in ganglion, spleen, lung, kidney and liver (Fig 6A). GFP-CD105^+^-MSCs were positive for CD105 and CD90. The results indicated that SDF-1 positive cells were co-localizing with GFP-CD105^+^-MSCs (Fig 6C).

## Discussion

Our group developed an efficient and reproducible procedure for the isolation of MSCs from synovial tissues of normal and osteoarthritis (OA) human donors based on the expression of the antigen CD105 and we demonstrated the cellular subset CD105^+^-MSCs posse chondrogenic capacity and also we demonstrated the similarity between CD105^+^-MSCs cultured from normal and OA synovial membranes reflected an absence of any effect from this pathological condition[1]. MSCs are the cell type of choice for articular cartilage tissue engineering because of their ease of isolation and expansion. MSCs multipotential differentiation capacity, especially the condrogenic differentiation property, make them the ideal candidate in cell therapy to replace the diseased skeleton like a joint disease [12, 13]. Moreover, MSCs contrasted immunomodulatory and anti-inflammatory functions make them the perfect candidate for cell therapy to treat diseases like OA and AR with inflammatory features [14], although research in this area is just starting to gain momentum. Human MSCs have engrafted and demonstrated site-specific differentiation after xenogenic transplantation into the joint sheep [15] and their migration and distribution when were transplanted into striatum of young Macaca fascicularis model, as described in detail previously Chen et al. [16]. It was treated to use the less number the animals to check where MSCs were migrating through circulatory system after their two ways injection. Two animals per group is the least number of individual to do a significant statistically comparision between median deviation when it is done combinations between injection method groups in front of cells labelled with different dyes and fluorescence. Therefore, MSCs are actively being considered as candidate cells for the treatment of arthritic joint diseases both as a structural substitute and as a stand-alone cell therapy or as a combination thereof[17, 18]. Current treatment for OA presents limited success focused to relieve pain temporarily. However, all of them presents limited success and includes several surgical procedures like debridement, drilling, osteochondral transplantation and autologous chondrocyte implantation Research into articular cartilage is a relatively recent effort and we still have a lot to learn about the normal development of the synovial joint and its components and their interaction in osteoarthritis and focal cartilage defects[18]. Our results obtained by biochemistry and haematological analysis indicated no stem cells transplantation-related toxicity was found in this study (Tables 2 and 3) similar results obtained by different types of analysis were published by Wang et al. [19].

Karlsson et al. [20] have demonstrated that a population of progenitor cells was present in the perichondral groove of Ranvier as well as within the articular cartilage in the knee, demonstrating their stem cell niche properties. However that population did not spontaneously self-repair an articular cartilage superficial lesion. Grogan et al. [21] showed in their last paper that abundant cells expressed accepted progenitor cell markers in human OA cartilage in front of normal one. However, the percentage of stem cells was much lower and within the range of their expected frequency. Alternatively, their increased expression in OA cartilage implicated an abnormal cell activation and differentiation development characteristic of OA. We found the same results in the OA knee from our animal model where found out there were significant statistically MSCs from the own animal in the injured areas (Fig 3C and 3D; Fig 4A, 4D and 4E). Several groups had published that increasing number of MSCs into OA cartilage could be because of cartilage degeneration coinciding with high bone turnover rates in vitro [22–25]. However, all those results already published must to be considered orientative respect to our data because the animal model used is different as well as the MSCs sources.

Isakova et al. [26] injected bone marrow-derived MSCs into young adult rhesus macaques´ CNS to evaluate their safety and practicality as vectors for direct intervention of neurologic disorders. Double labelling of sections confirmed that inserted donor cells lacked expression of the macrophage marker CD68. We did the same analysis confirming that the DiO-CD-105^+^- MSCs found in OA joint were negative for CD68 stain (Fig 5A).

SDF-1 is a powerful chemo-attractant cytokine that promotes directional migration of hematopoietic and non-hematopoietic cells[7, 27, 28]. We wondered what could be recruiting these cells only in the injured joints and not in the healthy ones, for answering this question we checked the expression of SDF-1 in the tissues where DiI-CD-105^+^- MSCs, DiO-CD-105^+^- MSCs (Fig 5B and 5C) or GFP-CD-105^+^- MSCs (Fig 6C) labelled cells were found. We observed that SDF-1 expression in the injured tissues was statistically significant higher with respect to healthy ones, it could be indicating that SDF-1 could be recruitment our labelled MSCs (Fig 5C) and also could be recruiting naive MSCs from periosteum to bone repair sites as published Kitaori[29] These data were similar to published with other authors where found increasing of expression of this cytokine which had a key role in the homing of hematopoietic cells to marrow in carcinomas which had a remarkable tendency to invade bone [30].

The method used to label the MSCs does not influence the results obtained because of not differences were observed between the MSCs labelled using vital dyes, DiI or DiO, with the MSCs transfected with GFP.

Labelled cells injected in the organism were not found in the healthy knee and nor tumours were found in any organ checked, it could be because of the sort time waited until sacrifice the animals, only one month, for this reason experiments longer than these must be done to discard tumour genesis and to check regeneration in the injured knee.

## Conclusions

Our results indicate that labelled synovial membrane MSCs are recruited by the injured joint directly from blood stream as well as cells injected directly in the injured joint keep there and they are able to go out from the normal knee along the time. We also demonstrated that labelled DiO-CD105^+^-MSCs, DiI-CD105^+^-MSCs and GFP-CD105^+^-MSCs keep the same cell type after the engraftment using immunofluorescence technique to test markers of mesenchymal stem cells which had been expressing in the cells previously to the injection like CD44, CD90 and CD105. Native cells positives for MSC-markers are mobilized in the injured joint in front of healthy one and no necessary because of the MSCs injected to help repairing the lesion. Although no tumours were found in any organ checked in the experimental animals after sacrificing, more long experiments in the time must be done.

### Ethical approval and consent to participate

All human tissues were obtained with fully informed consent from all subjects and ethical approval under the supervision of Ethical Committee of Clinical Investigation of Galicia. All the methods were carried out in accordance with the approved guidelines of Spanish law (14/2007 and 1716/2011) of Spanish Biomedical Investigation.

All the methods were carried out in accordance with the approved guidelines of Spanish law (1201/2005). All experimental protocols were approved by Animal Ethical Committee of Galicia.

## Supporting information

S1 Table NC3Rs ARRIVE gidelines checklines checklist-def.pdf. (PDF)

## Abbreviations

ALT: Alanine transaminase
AST: Aspartate aminotransferase
CD105^+^-MSCs: CD105^+^ enriched population of mesenchymal stem cells
DAPI: 4´,6´-diamidino-2-phenylindole
DiI: indocarbocyanine
DiO: oxacarbocyanine
FITC: fluorescein isothiocyanate
GFP: green fluorescence protein
OA: Osteoarthritis
PE: phycoerythrin
PE-Cy5: phycoerithrin cyano dye 5 conjugated
SDF-1: stromal cell-derived factor 1
WBC: white blood cells
